# Organic Solvent and Surfactant Resistant Paper-Fluidic Devices Fabricated by One-Step Embossing of Nonwoven Polypropylene Sheet

**DOI:** 10.3390/mi8010030

**Published:** 2017-01-22

**Authors:** Joong Ho Shin, Juhwan Park, Je-Kyun Park

**Affiliations:** Department of Bio and Brain Engineering, Korea Advanced Institute of Science and Technology (KAIST), 291 Daehak-ro, Yuseong-gu, Daejeon 34141, Korea; stvshn32@kaist.ac.kr (J.H.S.); juhwanpark@kaist.ac.kr (J.P.)

**Keywords:** embossing, organic solvents, paper-based fluidics, polypropylene sheet, surfactants

## Abstract

In this communication, we report a physical method for the fabrication of organic solvent and surfactant-resistant barriers on paper-based fluidic devices. When nonwoven polypropylene sheet is embossed with a steel mold, the embossed region acts as a physical barrier that can prevent the flow of liquids. Embossed polypropylene barriers not only block water, but also block organic solvents and surfactants, which are known to be difficult to handle on previous paper-based devices. Various amounts of embossing pressures were tested to determine the minimum embossing pressure required for leakproof barrier formation. The compatibility of the barrier was also investigated with several surfactants and organic solvents. As a demonstration, a lysis buffer, which was known to leak through wax-printed barriers, was used to detect *Escherichia coli* (*E. coli*) O157:H7. To the best of our knowledge, this paper is the first to report a one-step fabrication method of paper-fluidic devices capable of handling surfactants and organic solvents, including alcohols.

## 1. Introduction

Since their emergence, microfluidic paper-based analytical devices (μPADs) have gained a significant amount of attention and are growing rapidly. A distinguishing feature of a μPAD compared to conventional lateral flow assays is its ability to control the direction of fluid flow in the device, which is usually achieved by forming barriers that guide the fluid flow. In recent years, various methods have been developed to create barriers on many types of paper materials, and new methods are continuously being developed to improve the fabrication process of μPADs. A majority of publications involved the formation of hydrophobic barriers by embedding hydrophobic materials such as photoresist [1,2,3], wax [4,5,6] or parafilm [7,8] into various paper substrates, including nitrocellulose (NC) membrane, fabric, and chromatography paper. Fluidic channels can also be formed by printing hydrophobic agents [9,10] on hydrophilic substrates or etching agents on hydrophobic substrates [11]. Other creative methods were developed using a knife plotter to cut the papers into specific shapes [12]; a laser to hydrophillize a hydrophobic substrate [13], to polymerize pre-impregnated photopolymers in the NC membrane to create hydrophobic barriers [14], or to pattern hollow microstructures to form barriers [15]. From the many methods of barrier formation, wax printing on chromatography paper is currently one of the most widely used and most promising fabrication methods. However, it has been reported that wax-printed devices are not capable of handling surfactants and organic solvents [4].

One of the biggest disadvantage of wax-printed devices is that they cannot handle surfactants and organic solvents which can dissolve wax. However, surfactants are frequently used in biological assays, and being able to use them on paper-based devices can open up new possibilities and applications. Lysis buffers, for example, are required to release enzymes from bacteria, which can be used for colorimetric detection via substrate color change [16,17]. However, because the lysis buffer contains surfactants which can leak through wax barriers, novel methods are developed to contain such reagents. Hydrophobic silicone resin barriers [18] and hydrophobic sol–gel derived methylsilsequioxanes (MSQ) [19] have demonstrated effective containment of various reagents, including cell lysis buffer. However, alcohols such as methyl alcohol (methanol) and isopropyl alcohol (IPA) are not compatible with the proposed methods. Chemical-based barrier formation methods cannot be compatible with organic solvents if the solvents can dissolve the barriers. Moreover, the methods require multiple passes of printing for barrier patterning as well as having to print on both sides of the paper substrate, which can complicate the process.

In this communication, we report a paper-fluidic device capable of withstanding surfactants and organic solvents, including the aforementioned alcohols. The material used in this study is nonwoven polypropylene (PP) sheet, which is widely used to make everyday products such as diapers, water filters and wipers. PP itself is widely used to make objects that need to be resistant to chemicals and solvents such as pipette tips and tubes, and thus a paper fluidic device made of nonwoven PP sheet can also benefit from such properties. In our previous study, nonwoven PP sheet [20] and NC membrane [21] were used to create fluidic channels that allow fluid flow control. The surface of the PP-based device was pressurized to reduce the pore size and permeability of the sheet, and the flow rate control for the sequential delivery of multiple fluids was demonstrated. In this study, the pressure applied on the surface of paper was high enough to mechanically emboss the PP fabric and form a physical barrier through which liquids cannot penetrate. Conventionally, embossing of a nonwoven PP sheet is performed using two rolls of cylinders, whose surfaces are patterned so that the protruding surfaces can be used to press and emboss the fabric. Although the process is usually performed by cylinders that are heated above the melting point of the polymer, we present here that it can also be performed without heat to create a hydrophobic barrier.

## 2. Materials and Methods

### 2.1. Surfactant and Solvent Resistant Barrier Fabrication

Metal wires (diameter = 0.7 mm) were bent into specific shapes and used to create molds for the barriers. To create a barrier, a wire mold was placed on the top of PP sheet (Sunjin Industry, Nonsan, Korea) and pressed until the fibers were bonded to each other. To provide a flat surface on which the sheet can be embossed, the PP sheet was sandwiched between the metal wire mold and two acrylic plates. The stack was then placed on the top of a load cell (CLS-1 T; Curiosity Technology, Paju, Korea), and pressed together using a hand press machine (SWP-HP180-120S; Sam Woo, Siheung, Korea).

### 2.2. Determination of Minimum Pressure Required to Create a Leak-Proof Barrier

To determine the minimum pressure required to form a leakproof barrier, a PP strip (width = 5 mm; length = 2.5 mm) was pressed at the middle with a straight metal wire at varying pressure. The strip was suspended between two rubber spacers (Figure S1) to minimize liquid–surface contact, and 15 μL of blue dye (enough volume to wet the whole strip) was loaded on one end of the strip to observe leakage. Three strips were tested with each pressure and the pressure with which none of the three tested strips showed leakage was selected as the minimum pressure required to form a leakproof barrier.

### 2.3. Assessment of Barrier Resistance to Surfactants and Organic Solvents

Blue dyes were mixed with IPA, methanol, acetone, Triton X-100 and lysis buffer for visualization. A rectangular confinement (3 mm × 15 mm) was made on PP sheet by embossing, and the tip of the sheet was dipped into various solutions and allowed to wick upward. Rectangular confinements (barrier width = 1.5 mm) were also wax-printed on chromatography paper (Whatman No. 4) and heated at 150 °C for 3 min.

### 2.4. Bacteria Detection

Lysis buffer was made in the laboratory. The buffer consists of 1 mM ethylenediaminetetraacetic acid (EDTA), 1 mM Triton X-100, 150 mM NaCl, and 150 mM sodium dodecyl sulfate (SDS) in 10 mM Tris-HCl buffer. The buffer is known to lyse cells by solubilizing the cell membrane. *E. coli* O157:H7 (ATCC 35150) was grown in tryptic soy broth (211825; BD Biosciences, Seoul, Korea). For the demonstration of bacteria detection, bacteria were centrifuged and resuspended in the lysis buffer and mixed. 8 μL of solution containing 10^9^ CFU/mL *E. coli* O157:H7 was mixed with 2 μL of 20 mM chlorophenol red-β-d-galactopyranoside (CPRG) and the mixture was loaded into the test zones. The device was incubated for 1 h in a moist chamber to minimize evaporation during color development, allowing the sample to remain wet throughout the entire incubation time. *E. coli*’s intracellular enzyme β-galactosidase breaks down the CPRG and turns its color from yellow to magenta, indicating the presence of the bacteria.

## 3. Results and Discussion

**Figure 1:**
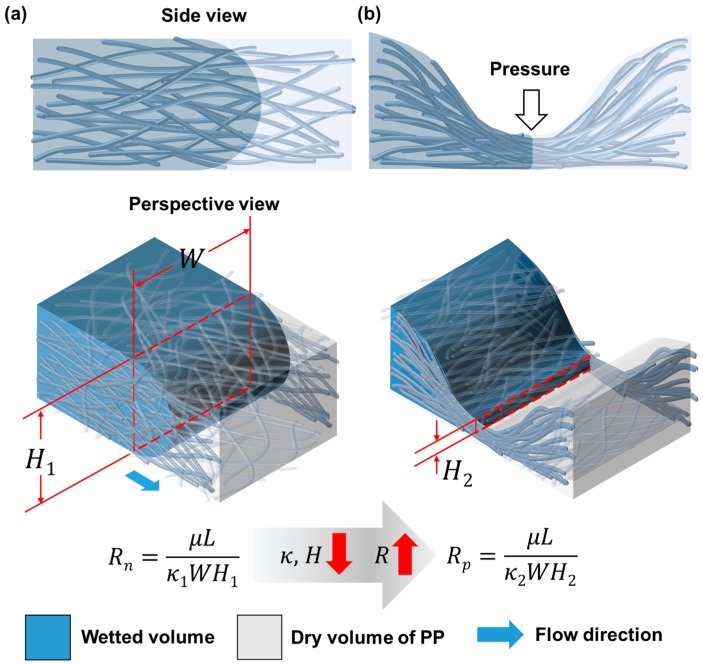
Working principle of an embossed barrier. Schematic representation of the side view and the perspective view of (**a**) liquid flowing through a PP sheet; and (**b**) liquid being blocked at an embossed region of the PP sheet.

Nonwoven PP sheet is composed of PP fibers that form a medium through which fluid can flow (Figure 1a). The flow through a PP sheet having a constant cross sectional area can be simplified by the Darcy’s law and is expressed by the following equation:

(1) $$Q=-\frac{\kappa \mathrm{WH}}{\mu L}\Delta P,$$

where *Q* is the flow rate, κ is the permeability of the sheet, *W* is the channel width, *H* is the channel height, μ is the viscosity of the liquid and *L* is the length of the channel. The term μL/κWH can be regarded as a resistance by comparing the equation with the Ohm’s Law, where *Q* is equivalent to the current, Δ*P* is equivalent to the voltage, which means that the resistance can be increased by decreasing the channel height and permeability. When sufficient pressure is applied to reduce the channel height and permeability and the resistance becomes high enough, the flow can be stopped (Figure 1b).

**Figure 2:**
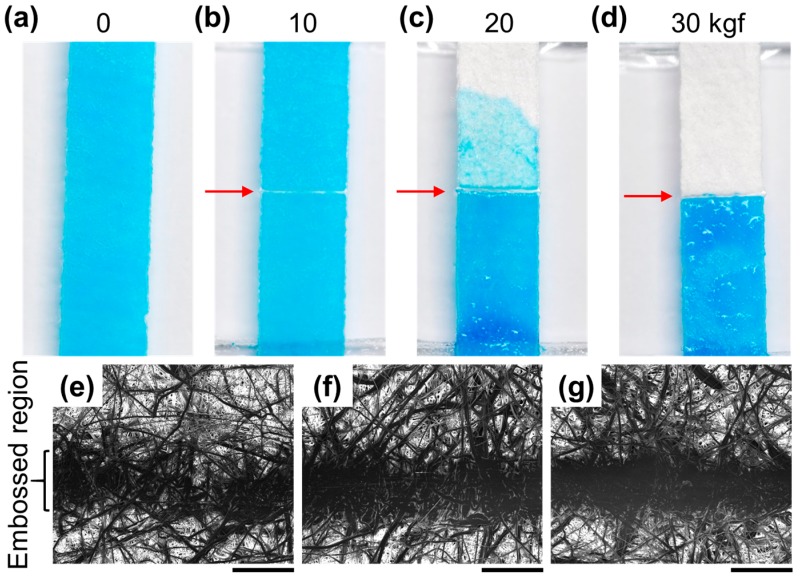
Embossed barrier assessment. (**a**) Distilled water mixed with blue dye was loaded to one end of the strip and wets the whole strip; (**b**) Strip embossed with 10 kgf; and (**c**) 20 kgf fails to prevent the dye solution from leaking; (**d**) Strip embossed with 30 kgf successfully prevents water from flowing over to the other side. Red arrow indicates the location of embossed barrier; (**e–g**) SEM images of the barrier embossed with 10, 20, and 30 kgf, respectively (scale bars = 200 μm).

Various amounts of embossing pressure were applied to the sheet in order to determine the minimum embossing pressure required to prevent flow. As shown in Figure 2a–d, pressing the strip with 30 kgf creates a leakproof barrier. The mechanical pressure delivered to the sheet is proportional to the force applied and inversely proportional to the area over which the objects contact the sheet (*Pressure = Force/Area*). Considering the width (200 μm) of the pressed region and the distance over which the paper was pressed (5 mm), we calculated the minimum pressure required to create the barrier as 294 MPa. Scanning electron microscope (SEM) images were compared for strips that were pressed with different forces. As the applied force increases, the embossed surface flattens to a greater extent and the gap between the fibers start to disappear (Figure 2e–g). Additional experiments were performed to assess the fluidic channel resolution of the embossed device. An embossing mold was fabricated by laser patterning a poly(methyl methacrylate) (PMMA) and the width of the smallest functioning fluidic channel was measured to be 285 μm (Figure S2).

**Figure 3:**
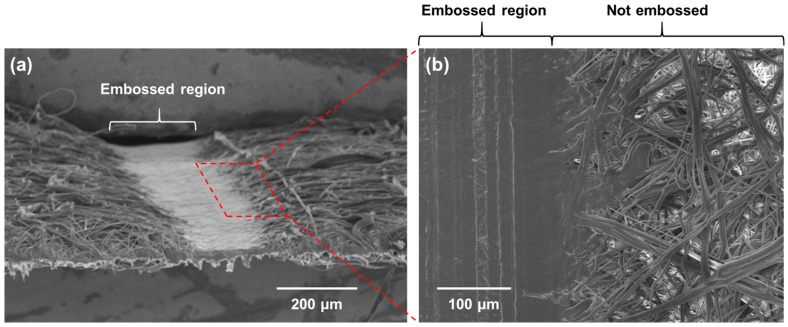
Close-up SEM images of embossed region. (**a**) Perspective view showing the difference in the sheet thickness; (**b**) Close-up view of the top of an embossed region showing flattened and densely packed fibers, with no apparent gaps between the fibers.

A perspective view of the embossed region (Figure 3a) shows the flattened surface of the embossed region and a decreased thickness. A top view of the embossed region (Figure 3b) shows that the gaps through which liquid flows via capillary action are physically closed off due to the embossing process, thereby creating a leakproof barrier. Compared to fiber network of a normal PP sheet, the fibers of the embossed region are deformed, flattened and densely packed with each other. Such modification in the physical characteristics of the sheet can occur because PP fibers, which consist of both an amorphous phase and a crystalline phase, undergo plastic deformation [22]. It is important to note that the embossing method is not limited to creating linear barriers. Various mold shapes can be easily fabricated, for example, by laser patterning a PMMA, and can be used to emboss PP-based devices of various patterns (Figure S3).

**Figure 4:**
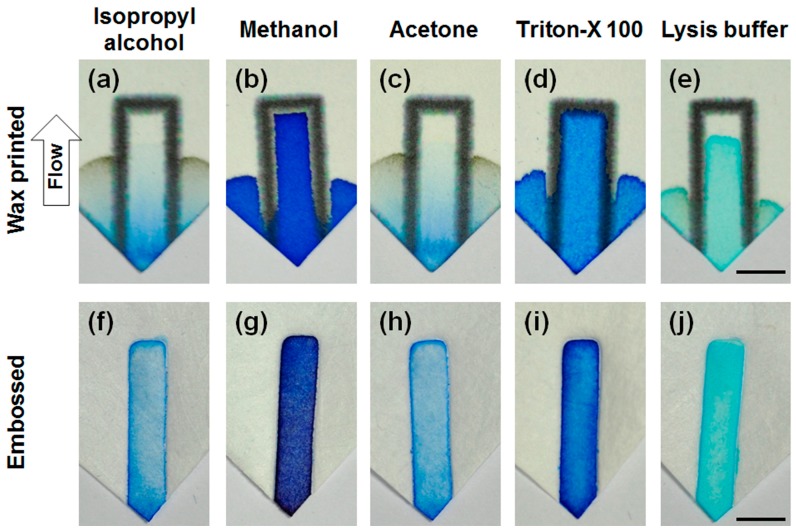
Leakage test with organic solvents and surfactants. (**a**–**e**) Wax-printed chromatography papers failed to contain the reagents within the barrier; (**f–j**) embossed PP successfully contained all of the reagents tested (scale bars = 5 mm).

The embossed PP barrier’s compatibility with surfactants and organic solvents were tested. A rectangular confinement was made on PP sheet, and the tip of the sheet was dipped into various solutions and allowed to wick upward. Figure 4 shows that wax-printed barriers on chromatography paper is incapable of containing any of the tested reagents as mentioned in previous studies. On the other hand, embossed barriers on PP sheet are able to withstand IPA, methanol, acetone, Triton X-100 and lysis buffer. The result is significant considering the fact that alcohols such as IPA and methanol were not able to be contained by silicone printing [18] nor MSQ barrier [19]. PP sheet with embossed barrier can contain such reagents by bonding the fibers together and physically blocking the fluid path as observed by SEM. Furthermore, we confirmed that PP is chemically more resistant than NC membrane by exposing them to acetone and dimethyl sulfoxide (DMSO), which are known to dissolve the NC membrane [23]. While the NC membrane could be dissolved by both acetone and DMSO, the solvents had no adverse effects on the PP strip and did not leak through the embossed barrier (Figure S4).

**Figure 5:**
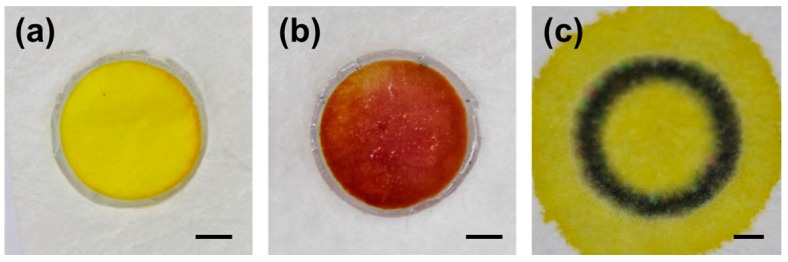
*E. coli* O157:H7 detection result. (**a**) Negative; and (**b**) positive control samples are loaded into embossed barriers, and are contained without leaking. The distinct color change indicates the presence of bacteria; (**c**) Loaded sample breaches through the conventional wax-printed barrier (scale bars = 1 mm).

To demonstrate possible applications of the surfactant-resistant barrier, a bacteria sample was detected on an embossed PP test zone. As mentioned previously, the enzyme-based bacteria detection involves lysis of the cells using lysis buffer, which contains surfactants that solubilize the cell membrane and release its internal enzymes. *E. coli*’s intracellular enzyme β-galactosidase breaks down the CPRG and the change in its color from yellow to magenta indicates the presence of the bacteria. Sample solutions containing bacteria mixed with lysis buffer and CPRG were loaded into the test zones. Figure 5a,b shows that both negative and positive control samples are contained within the embossed barrier without any leakage and the presence of bacteria can be distinguished by the color change. On the contrary, as reported previously, the sample breaches through the wax-printed barrier (Figure 5c), which is detrimental to analysis [18,19]. This result shows that an embossed barrier is compatible with surfactant-based buffers for bioassay applications and also has the potential to be used to create arrays of paper-based microzones [24]. It is worth noting that the samples remained wet within the test zone throughout the entire incubation time without leaking, indicating that the barrier does not leak over time.

## 4. Conclusions

A paper-based device’s barrier that is resistant to organic solvents and surfactants is introduced. The fabrication involves embossing of a nonwoven PP sheet, which can be done in one step. SEM images reveal that the barrier is formed by physically bonding the PP fibers together and closing off the gaps through which liquid can flow. The minimum embossing pressure required to create a leakproof barrier is determined to be 294 MPa. The organic solvent and surfactant compatibility of the embossed barrier was compared with wax-printed barriers, and the embossed barrier is able to contain all of the reagents including IPA and methanol, which could not previously be handled on paper-based devices. PP is also chemically resistant to acetone and DMSO, which are known to solubilize the NC membrane. As a demonstration, bacteria detection was performed on embossed test zones and a sample containing lysis buffer was successfully contained within the test zones without leakage, allowing a clear interpretation of the colorimetric assay results. The main advantage of embossing is that it is a one-step process and highly suitable for high-throughput mass production. The method is compatible with the roll-to-roll fabrication process, which can be realized by using patterned cylindrical rollers for embossing and continuously producing paper-based devices. In contrast to chemical barrier-patterning methods that involve the embedding or depositing of hydrophobic materials into the paper substrate, the embossing method does not require any additional material for the formation of barriers, which can also lower the manufacturing cost in the mass production scale. Currently, the most widely used materials for paper-based devices are chromatography paper and NC membrane. This paper is the first paper to explore the possibility of using PP as a robust material that is compatible with organic solvents and surfactants. Because PP is a material well known for being resistant to almost all organic solvents, we expect that the use of nonwoven PP sheet as a substrate can broaden the range of applications and also offer new opportunities in the field of paper-based fluidics.

## Supplementary Materials

The following are available online at www.mdpi.com/2072-666X/8/1/30/s1, Figure S1: Setup for PP strip suspension, Figure S2: Embossed channel resolution test, Figure S3: Pictures showing examples of embossed PP-based devices before and after the loading of color dyes, and Figure S4: NC membrane solubility in solvents.
