# Supplementary material for: Organic Solvent and Surfactant Resistant Paper-Fluidic Devices Fabricated by One-Step Embossing of Nonwoven Polypropylene Sheet
Source: Micromachines (Basel). 2017 Jan 22;8(1):30. doi: 10.3390/mi8010030 (PMC6190025; doi:10.3390/mi8010030)
Supplement: Supplementary file 1 [file micromachines-08-00030-s001.pdf]

Supplementary Materials: Organic Solvent and Surfactant Resistant Paper-Fluidic Devices Fabricated by One-Step Embossing of Nonwoven Polypropylene Sheet

Joong Ho Shin, Juhwan Park and Je-Kyun Park

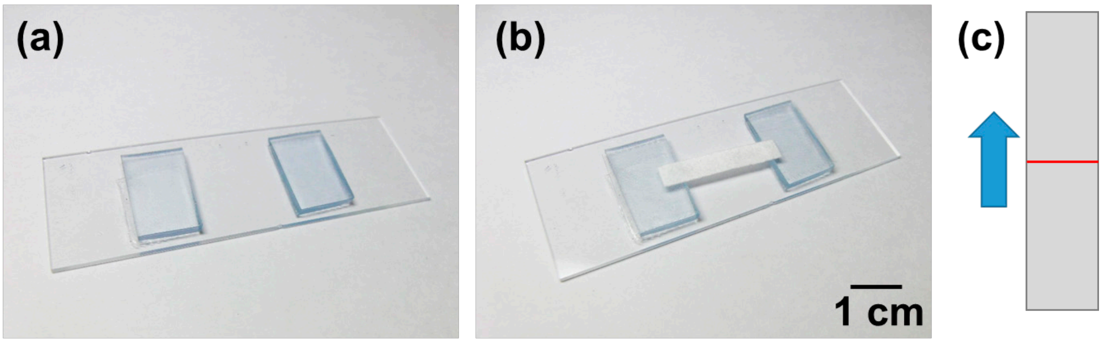

Figure S1. Setup for polypropylene (PP) strip suspension. (**a**) 1.25 mm thick rubber pads were placed on a glass slide; (**b**) PP strip was suspended on the rubber pads with double sided tapes. Suspension was provided to prevent the liquid's interaction with the glass surface; (**c**) Schematic of the strip for barrier formation experiment. Red line indicates the location of embossed barrier and the arrow indicates the direction of flow after loading dye.

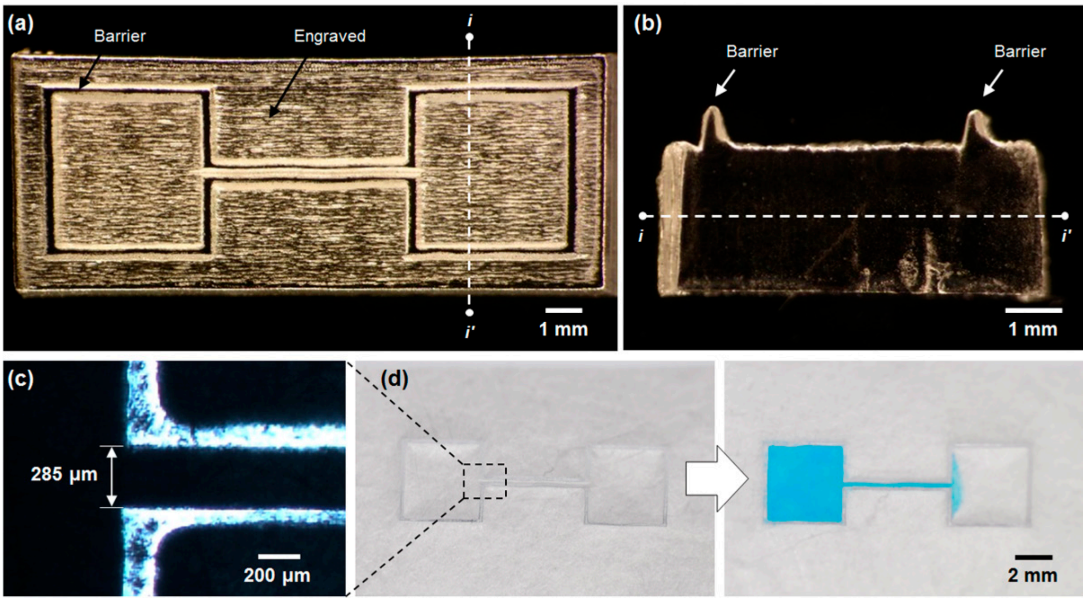

Figure S2. Embossed channel resolution test. Dark field images showing (**a**) a top view of the laser patterned poly(methyl methacrylate) (PMMA) mold; and (**b**) a cross sectional view of the mold section. Laser cutter's engraving mode was used to create a standing barrier by ablating the surface of PMMA while leaving the barrier part protruding out of the mold's surface (A section of the mold was cut out using a laser cutter to show the protrusion); (**c**) A bright field image showing the channel entrance portion of the embossed PP-based device; (**d**) Photos showing the embossed device before and after loading the dye. The loaded dye is able to flow through the channel to the other side without leaking.

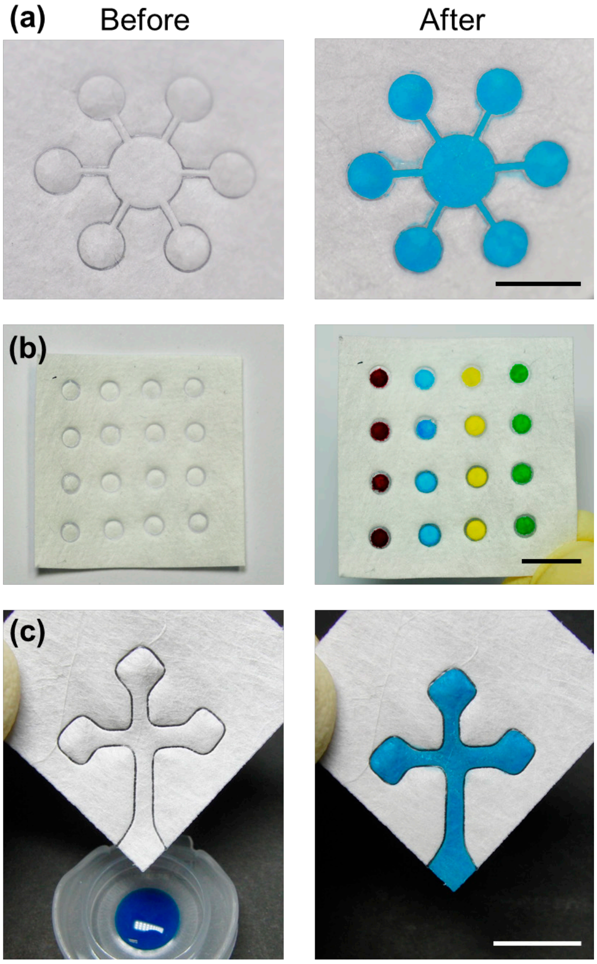

Figure S3. Pictures showing examples of embossed PP-based devices before and after loading color dyes. (**a**) A device with multiple test zones (scale bar = 5 mm); (**b**) An array of microzones (scale bar = 1 cm); (**c**) A three-channel device for dipping (scale bar = 1 cm).

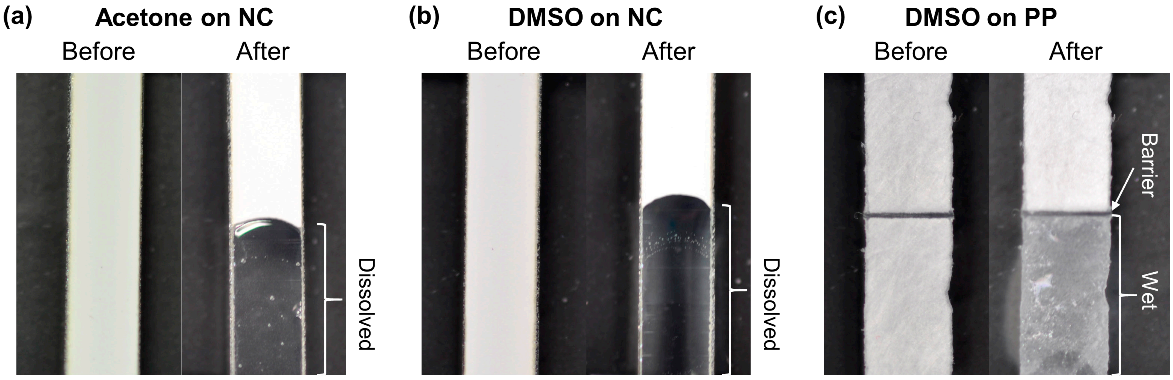

Figure S4. Nitrocellulose (NC) membrane's solubility in organic solvents. Pictures show the strips before and after loading the solvents. (**a**) Acetone; and (**b**) dimethyl sulfoxide (DMSO) dissolves NC membrane; (**c**) PP membrane does not dissolve with DMSO, and its barrier is able to withstand the organic solvent without leaking.
